# Supplementary material for: Distinctive pupil and microsaccade-rate signatures in self-recognition
Source: J Vis. 2025 Apr 29;25(4):16. doi: 10.1167/jov.25.4.16 (PMC12045120; doi:10.1167/jov.25.4.16)
Supplement: Supplement 1 [file jovi-25-4-16_s001.pdf]

A. Supplemental Online Materials

A.1. LMM Random Effects Structure

We used linear mixed models (LMMs) to analyze the pupillometry data in 5 time windows. In all time windows we employed the same fixed and random effect structure. Random effects were estimated for both participants and images, but mainly with the aim of correcting for their influence. We initially formulated a hypothesis-driven random effect structure, including random intercepts for both participants and images, and random slopes for each the Faces comparisons. We included the Repetition comparisons as random slopes for each subject (but not each image, as not all images were necessarily seen in each repetition). We also excluded random interaction slopes. Model reduction was then performed iteratively until convergence was achieved without issues. Specifically, correlation terms were first removed, followed by the least varying random effect terms. In cases where two converging models with different random effect terms were obtained, we used Bayesian-Information-Criterion (BIC) to select the best model. To ensure that none of the models were degenerate, a principal component analysis was performed on the random effect terms (Bates et al., 2015). The final model structure is as follows,

$$+(1 + Face + Rep2 + Rep3|VP) + (1 + Face(Se - O)|Img). \tag{6}$$

We performed the same procedure for the random effects structure of the explorative LMM that explores the relationship between microsaccade occurrence and pupil size. Here we included random intercepts by subject. As the predictor variable was ad-hoc, group sizes were uneven and a random effect of image was excluded. After the model reduction procedure we arrived at a model which defines only a random intercept for each subject.

$$+(1|VP). \tag{7}$$

A.2. Controlling for Knowing

After the experiment, participants were shown all faces one more time. They were asked to indicate by pressing one of 3 buttons on a *ViewPixx* button box whether they know the person in the picture, did not know them, or know them very well. With this information we can exclude trials with subjects who coincidentally did know each other across the schools. Figure A1 shows a visualization of the social network between the participants. This data also allows potential exploratory analyses using the distinction of close peers versus acquaintances.

A.3. Controlling for Self Image

For the purposes of an exploratory analysis, we asked three questions that were to be answered on a 5 point scale, i.e.,

1. How did you like the photographs in general? *original: Wie fanden Sie die Fotos allgemein?*
2. How did you like the photograph of yourself? *original: Wie fanden Sie das Foto von sich selbst?*
3. How happy are you with your own appearance in general? *original: Wie glücklich sind Sie mit Ihrem eigenen Erscheinungsbild allgemein?*

Questions 1 and 2 were to be answered with

1. Liked very much *original: Sehr gelungen*
2. Liked *original: Gelungen*
3. Neutral *original: Durchschnittlich*
4. Did not like much *original: Eher nicht gelungen*

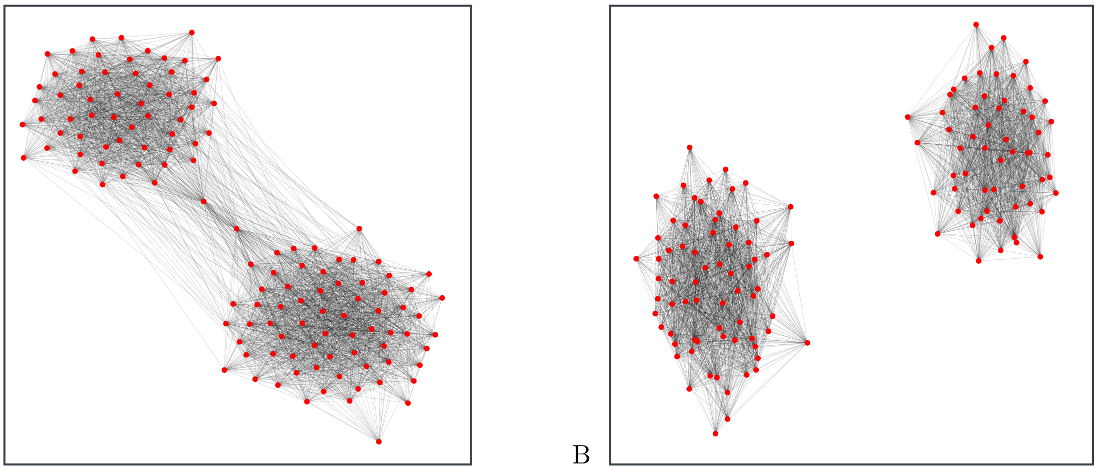

A B

Figure A1: **Social Network of participants.** The two clusters represent the two school classes. The right panel shows the social participants that knew each other. The left panel shows the connections after removing trials that violated the expectation that students only know people from their own class.

5. Did not like *original: Nicht gelungen*

and question 3 with

1. Very happy *original: Sehr glücklich*
2. Happy *original: Glücklich*
3. Neutral *original: neutral*
4. Rather unhappy *original: Eher unglücklich*
5. Unhappy *original: unglücklich*
